# *Lactobacillus acidophilus* and HKL Suspension Alleviates Ulcerative Colitis in Rats by Regulating Gut Microbiota, Suppressing TLR9, and Promoting Metabolism

**DOI:** 10.3389/fphar.2022.859628

**Published:** 2022-05-04

**Authors:** Kasimujiang Aximujiang, Kuerbannaimu Kaheman, Xilinguli Wushouer, Guixia Wu, Abulaiti Ahemaiti, Kurexi Yunusi

**Affiliations:** ^1^ Department of Biochemistry and Molecular Biology, School of Basic Medical Sciences, Xinjiang Medical University, Urumqi, China; ^2^ College of Pharmacy, Xinjiang Medical University, Urumqi, China; ^3^ Department of Rehabilitation Medicine, First Affiliated Hospital in Xinjiang Medical University, Urumqi, China; ^4^ Department of Biology, School of Basic Medical Sciences, Xinjiang Medical University, Urumqi, China; ^5^ Department of Physiology, School of Basic Medical Sciences, Xinjiang Medical University, Urumqi, China; ^6^ The Functional Center, School of Basic Medical Sciences, Xinjiang Medical University, Urumqi, China; ^7^ Uygur Medical College, Xinjiang Medical University, Urumqi, China

**Keywords:** ulcerative colitis, intestinal flora, metabolomics, HKL, *Lactobacillus acidophilus*

## Abstract

Ulcerative colitis (UC) is a chronic non-specific inflammatory bowel disease with complex pathogenesis. The intestinal flora disturbance affects the homeostasis of the intestinal environment, leading to metabolic imbalance and immune abnormalities of the host, contributing to the perpetuation of intestinal inflammation. We suggest that the combination of anti-inflammatory therapy and the regulation of intestinal flora balance may help in the treatment process. Previously, we used a combination treatment consisting of *Lactobacillus acidophilus* (Lac) and Chinese medicine Huan Kui Le (HKL) suspension in a UC rat model, where the combined intervention was more effective than either treatment alone. Herein, the mechanism of action of this combined treatment has been investigated using 16S rRNA sequencing, immunohistochemistry, and ELISA methods in the colon, and untargeted metabolomics profiling in serum. Colon protein expression levels of IL-13 and TGF-β were upregulated, whereas those of TLR9 and TLR4 were downregulated, consistent with an anti-inflammatory effect. In addition, gut microbiota structure changed, shown by a decrease in opportunistic pathogens correlated with intestinal inflammation, such as *Klebsiella* and *Escherichia-Shigella*, and an increase in beneficial bacteria such as *Bifidobacterium*. The latter correlated positively with IL-13 and TGF-β and negatively with IFN-γ. Finally, this treatment alleviated the disruption of the metabolic profile observed in UC rats by increasing short-chain fatty acid (SCFA)–producing bacteria in the colonic epithelium. This combination treatment also affected the metabolism of lactic acid, creatine, and glycine and inhibited the growth of *Klebsiella*. Overall, we suggest that treatment combining probiotics and traditional Chinese medicine is a novel strategy beneficial in UC that acts by modulating gut microbiota and its metabolites, TLR9, and cytokines in different pathways.

## Introduction

Ulcerative Colitis (UC) is a refractory intestinal inflammatory disease and a subclass of inflammatory bowel diseases (IBD) (Mehandru and Allen, 2017). UC is mainly characterized by persistent inflammation of the intestinal mucosa or submucosa. The main clinical symptoms include diarrhea, abdominal pain, weight loss, intestinal bleeding, and blood in the stool (Gong et al., 2015). In recent years, especially in the past few decades, UC incidence worldwide has been on the rise in developing countries (Kaplan, 2015; Kaplan and Ng, 2017; Mitchell et al., 2018; Schoultz and Keita, 2019). Its etiology and pathogenesis are still not clear, and there is no treatment that can completely eliminate its symptoms.

Traditional Chinese medicine (TCM) is widely used in the treatment of UC (Dai et al., 2017; Zheng et al., 2017; Cui et al., 2018). TCM drugs are closely associated with immunity, e.g., reducing pro-inflammatory cytokines (Liu et al., 2012; Zou et al., 2016). However, disruption of the intestinal mucosal barrier and abnormal activation of the immune system are associated with intestinal flora imbalance. Any change in the gut microbiota can lead to malnutrition and can exacerbate pathological changes such as chronic inflammation (McGovern et al., 2015; Luo et al., 2017). Therefore, in addition to anti-inflammatory treatment, UC therapy requires the application of microecological regulators to correct the imbalance of the intestinal flora.

Probiotics maintain the health of the intestinal tract of the host by regulating its intestinal flora, acting on the intestinal barrier, and regulating immunity (Liu and Zhang, 2015). The role of several probiotics in the treatment and prevention of intestinal infections has been identified in *Bifidobacteria, Enterococcus* and *Lactobacillus* (Kruis, 2004; Fung et al., 2011; Baumgart and Sandborn, 2012). *Lactobacillus* is the most common bacterium and is considered the most beneficial probiotic, as it has been shown to prevent intestinal diseases (Matur and Eraslan, 2012; Lee et al., 2017; Plaza-Diaz et al., 2017). *Lactobacillus* can reduce the expression of TLR and reduce intestinal inflammation (Lee Y. K. et al., 2009). In diarrhea mice, it can reduce the mRNA content of TLR2 and TLR4 while reducing the degree of bacterial translocation (Zhang et al., 2011).

A large number of differential metabolites produced in UC patients are closely related to the imbalance of intestinal flora (Wang et al., 2017). For example, the increase in the content of organic acids in urine indicates the overgrowth of *Bifidobacterium*, *Lactobacillus,* and rare *Micrococcus* in the intestine (Lord and Bralley, 2008; Nicholson and HolmesE, 2012). *Lactobacillus* produces organic acids, hydrogen peroxide, carbon dioxide, and other antimicrobial compounds that may inhibit potential pathogens (Lee J. H. et al., 2009; Patel et al., 2014). Flagellin and lipopolysaccharide produced by the intestinal flora regulate human fat metabolism through the nuclear factor interleukin-3 (IL-3) (Yun et al., 2017). Changes in the content of phenylacetic, phenyl propionic, succinic, hydroxyphenyl acetic, fumaric and 3-indoleacetic acids can be caused by the activity of *Clostridium* and *Bacteroides* bacteria involved in the metabolism of phenylalanine and tyrosine (Sitkin et al., 2014). The intestinal flora affects metabolism, and changes in metabolites can also affect the intestinal flora. Therefore, the study of the relationship between intestinal flora, immunity, and metabolism from a local and global perspective can be used to understand the mechanism of UC pathogenesis and its treatment with drugs. The combination of *Lactobacillus acidophilus* and HKL suspension in UC rats was more effective than either treatment alone (Kasimujiang et al., 2021). However, the mechanism of this combination therapy is not understood.

Herein, we have used *Lactobacillus acidophilus* and HKL in rats with TNBS-induced UC. A correlation between the composition of the intestinal flora using 16srRNA technology and parameters such as serum metabolites, TLRs, and cytokines were used to explore the occurrence and development of UC and the underlying mechanism of using a combined therapy. We provide an experimental basis for UC clinical treatment and for the determination of new therapeutic targets.

## Materials and Methods

### Experimental Drugs

*Lactobacillus acidophilus* was used as freeze-dried powder, with a total number of live bacteria of 1.44 × 10^10^ cfu/g. *Escherichia coli* MPN/g was ≤3.0, and water content was 5.7%. The strain composition was *Lactobacillus acidophilus,* and the carrier was isomalt oligosaccharide.

HKL suspension: HKL suspension prescriptions include Quercus infectoria galls (QIG), pomegranate flower, amber, Bambusae Concretio Silicea, Coptis chinensis Franch, Halloysitum rubrum, Polygonum bistorta, Sanguis Draconis, Cydonia oblonga, Plantaginis Herba, and rose dew. The suspension was prepared according to the prescription proportion before administration (Ayijiamali et al., 2020; Kasimujiang et al., 2021). (The preparation process of this drug has been authorized by China’s national invention patent. Patent Number: ZL201810790421.7). The main component in HKL is QIG. Research reports show that QIG is rich in tannins with about 50–70%, followed by gallic acid, ellagic acid, hexamethyl, syringic acid, amentoflavone, sitosterol, and glucose propionic acid. It has various pharmacological activities, including antifungal, antiviral, insecticidal, wound healing, gastric protective effects, and anti-inflammation (Zhang et al., 2020)

### Experimental Rats

Male Wistar rats (200–230 g) were purchased from the Experimental Animal Center of Xinjiang Medical University (Xinjiang, China) and were maintained in a cleanroom at 25 ± 3°C with 60–80% relative humidity, keeping a 12-h light/dark cycle, and acclimatized for 1 week. All the experiments were approved by the Ethics Committee of Xinjiang Medical University (Permit Number: IACUC20180814-15).

### Establishment of Ulcerative Colitis Rat Model and Drug Intervention

The TNBS-induced UC rat model was established as described (Morris et al., 1989). The rats were randomly divided into five groups, including the normal group (*n* = 10), UC group (*n* = 20), HKL treated group (*n* = 20), Lac treated group (*n* = 20), and Lac and HKL treated group (LH group, *n* = 20). After a 24 h fasting period with access to water ad libitum, the UC model was established in other groups except for the normal group. Then, TNBS (70 mg/kg) was dissolved in 50% ethanol, and the mixed solution was injected into the proximal end of the descending colon slowly using a 3-mm enema tube. The rats in the normal group received an injection of physiological saline. After two days, the rats in each group were subjected to drug treatment. The normal and UC groups were given sterilized water, 2 ml twice a day, whereas the other TNBS-treated groups were supplemented with either HKL (1.8 g/kg) suspension (HKL group), Lac (0.21 g/kg) (Lac group), or *L. acidophilus +* HKL (0.21 g/kg Lac + 1.8 g/kg HKL) (LH group). After 14 days of treatment, the rats were sacrificed under anesthesia, and the colon tissue (1.5–8.5 cm from the anus) and blood were collected.

### Hematoxylin and Eosin Staining

One part of the isolated colon was fixed in 4% paraformaldehyde for 2 h and embedded in paraffin. Colon tissue sections were prepared, stained with hematoxylin and eosin (H&E), and assessed under a light microscope. Pathologists described ulcer depth and inflamatory cell infiltration degree.

### ELISA Analysis

The levels of IL-4, IL-6, IL-10, IL-12, IL-13, IL-17, TGF-β, and IFN-γ in colon tissue samples were measured using ELISA kits (Genetic Beauty, Wuhan, China) according to the manufacturer’s instructions. The protein concentration measured in each sample is expressed in mg/L. The colon tissue weight of each sample was 50 mg, content = concentration/50.

### Immunohistochemical Staining

Colonic tissue sections underwent deparaffinization, rehydration, and washing with phosphate buffer saline (PBS) PBS. Sections were blocked with 10% goat serum and underwent successive incubations with primary (overnight, 4°C) and secondary (37°C, 30 min) antibodies. Then, diaminobenzidine and hematoxylin were used for counterstaining. The protein expression of TLR4 and TLR9 in colon tissue was observed and recorded under a microscope.

### Bacterial Genomic DNA Extraction

Genomic DNA was extracted from colon samples (*n* = 6/group) using the Fast DNA ®Spin Kit (MP Biomedicals, US). DNA yield was quantified using a NanoDrop-2000 spectrophotometer. DNA (10 ng) was used as a template in PCR amplification and further microarray analysis.

### Illumina Sequencing and Data Processing

The V3–V4 region of 16S rRNA was amplified for sequencing using standard protocols using the Illumina Miseq platform (PE 300; Major Bio-Pharm Technology Co. Ltd., Shanghai, China). Raw fastq files were demultiplexed, quality-filtered by Trimmomatic, and merged by FLASH. UPARSE software (version 7.0.1090; http://drive5.com/uparse/) was used to perform operational taxonomic units (OTUs) clustering on 97% similarity sequences. RDP classifier (version2.11; http://sourceforge.net/projects/rdp-classifier/) was carried out for species classification annotation for each sequence. The Silva database (version 132; http://www.arb-silva.de) was used for alignment, where the threshold was set to 70%.

### Bioinformatics Analysis of RNA Sequencing Data

Differences in α-diversity indices were tested using the Welch T-test (Mothur, v1.30.1, http://www. mothur.org/wiki/Schloss_SOP# Alpha _diversity). The Kruskal–Wallis H rank non-parametric test assessed differences in microbiota composition, as assessed by β-diversity metrics. Correlations between gut microbiota and cytokines, TLRs, or metabolites, were analyzed using an RDA/CCA test implemented in R 3.2.2 software (heatmap package). LEfSe (Linear discriminant effect size) analysis based on the non-parametric factorial Kruskal–Wallis test was performed using the default parameters from the phylum to the genus taxonomic level to identify microbial biomarkers in the gut microbiota of LH and UC groups. LEfSe used LDA (linear discriminant analysis) was used to estimate the effect of the abundance of each species on the observed effects. The threshold on the LDA score for discriminative biomarkers was 3.0. All statistical analyses were conducted using R 3.2.2.

### Blood Sample Preparation for Metabolomics Analysis

Before the metabolomics analysis by ^1^H NMR spectroscopy, 5 ml of abdominal aortic blood was taken and left at room temperature for 30 min, followed by centrifugation at 3000 rpm for 15 min at 4°C, and the supernatant was sub packed into Eppendorf Micro Test Tubes (EP tubes). After that, 200 µL of blood serum and 400 µL of phosphate buffer (sodium chloride, NaH_2_PO_4_, and K_2_HPO_4_ in ultra-pure D_2_O water, pH 7.4) were mixed in an EP tube and centrifuged at 10,000 rpm for 10 min at 4°C. Then, 550 µL of filtered serum was transferred to a 5 mm NMR tube. 1D proton spectra were acquired using VnmrJ software (Varian NMR Systems) and a Varian Inova ^1^H NMR spectrometer (Agilent Technologies Inc., Santa Clara), operating at 600 MHz and 298 K (25°C). The relevant parameters are frequency 599.95 MHz, pulse 8.2 µs, relaxation delay intervals 2 s, sampling points 64 k, 128 scans, and 16,384 data points.

### Spectrum Acquisition and Metabolite Quantification

MestReNova software was used to adjust the baseline and phase of the NMR spectrum, and the portion signal of the gluco-chemical site (5.233 ppm) was calibrated to eliminate the water peak between 4.66 and 5.20 ppm. In the range 0.5–10.00 ppm, each 0.003 ppm segment of the spectrum was segmentalized and normalized. After segmentation, integral values were imported into SIMCA-P+11statistical software for principal component analysis (PCA) and partial least squares-discriminant analysis (PLS-DA). Important metabolites were selected according to the VIP score derived from PLS-DA. Differential metabolites were identified based on criteria of VIP >1 and P(Corr) > 0.3.

### Statistical Analysis

All the analyses were performed using SPSS version 21. Continuous variables are presented as mean and standard deviation, whereas categorical variables are expressed as percentages. Baseline characteristics were compared using the Welch T-test or one-way ANOVA test (continuous variables). A Kruskal–Wallis H rank non-parametric test was used for non-normally distributed variables.

## Results

### *L. acidophilus* Enhances the Anti-Inflammatory Effect of HKL Suspension

**FIGURE 1:**
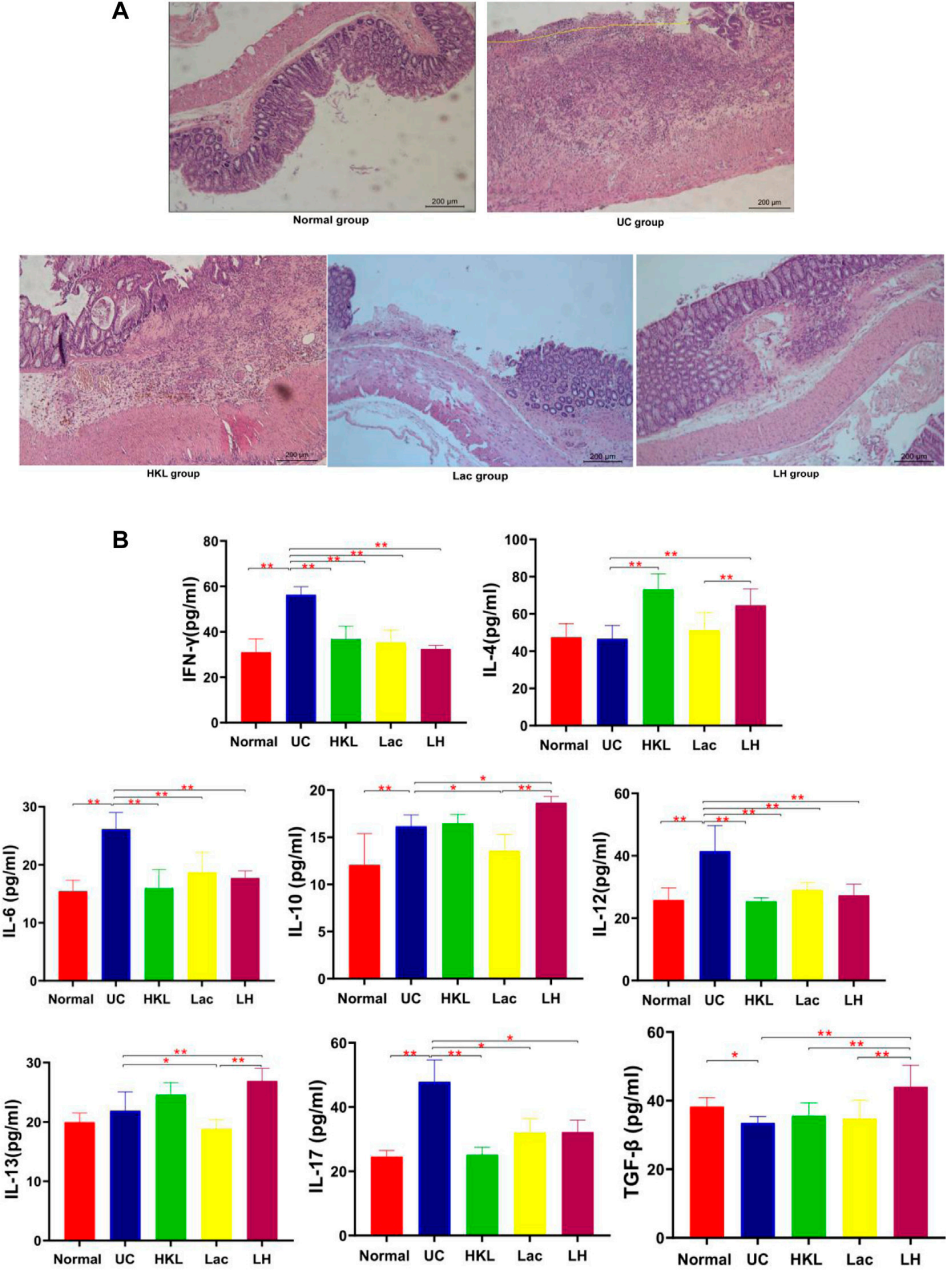
LH treatment ameliorated inflammatory infiltration and histopathological injury in TNBS-treated rats. **(A)** Hematoxylin and eosin staining of colon tissue (*n* = 8). **(B)** IFN-γ, IL-4, IL-6, IL-10, 1L-12, 1L-13, 1L-17, and TGF-β levels were determined by ELISA (*n* = 6).**p < 0.01,*p < 0.05 vs. UC group.

H&E staining (Figure 1A) showed that the UC group exhibited severe inflammation, cell infiltration, disruption of crypt structure, and loss of mucosal architecture and epithelium, ulceration of mucous, which suggested severe intestinal barrier damage. In LH rats, colonic mucosal glands were arranged regularly, in contrast to Lac and HKL groups, with some erosion but no apparent ulcer and only a small amount of inflammatory cell infiltration, mucosal epithelial cell proliferation, and granulation tissue formation. Since inflammatory cytokine expression plays an essential role in the pathogenesis of IBD (Soufli et al., 2016), we studied the effect of LH treatment on intestinal inflammation. Whereas in the UC group all pro-inflammatory cytokines (IL-6, IFN-γ, IL-12, and IL-17) were significantly increased compared to the normal group (Figure 1B), LH treatment reduced these levels and increased those of anti-inflammatory cytokines (IL-10, IL-13, and TGF-β). These results are consistent with an additive anti-inflammatory effect of *L. acidophilus* and HKL treatment (Figure 1B).

### *L. acidophilus* Enhances the HKL Inhibitory Effect on TLR4 and TLR9

**FIGURE 2:**
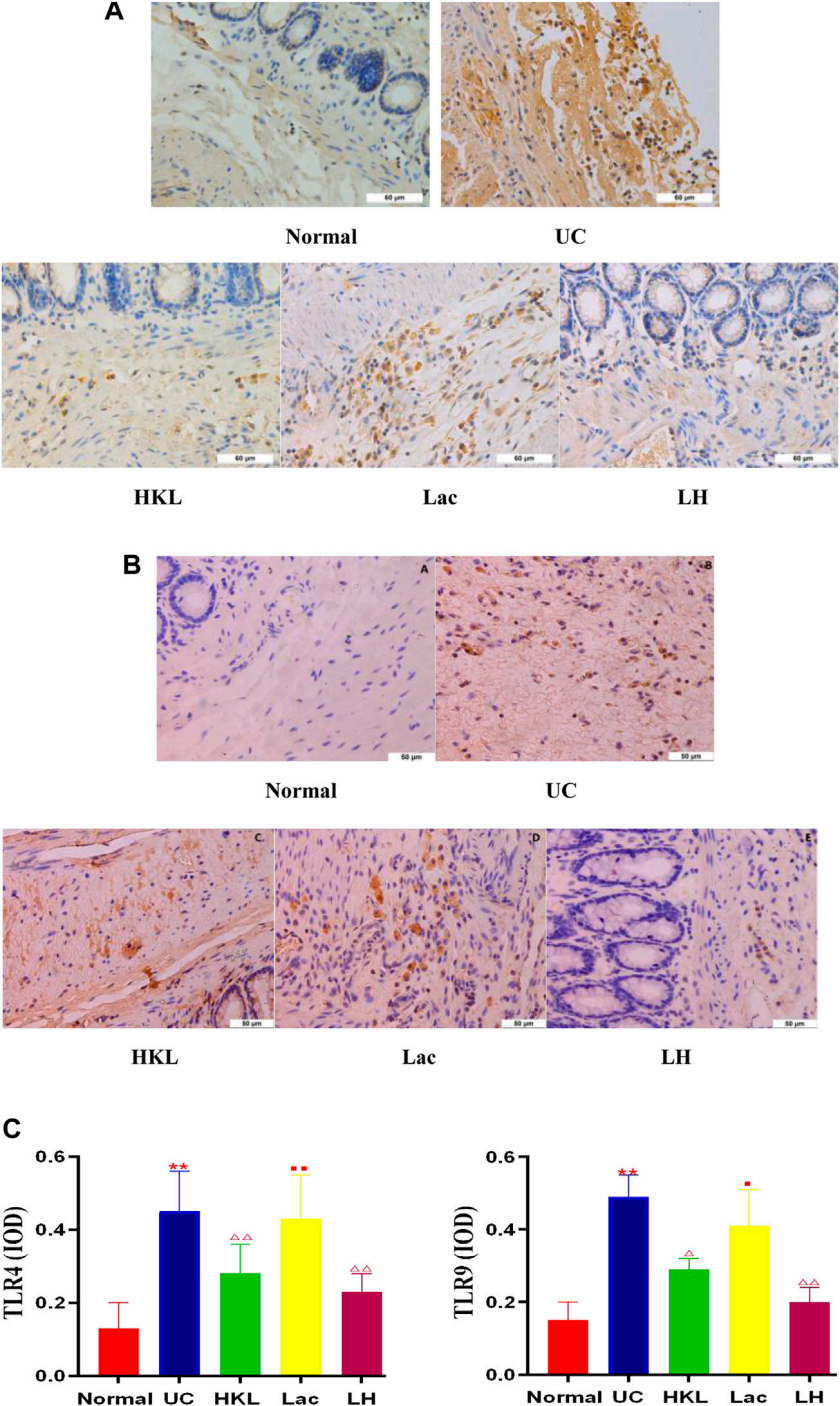
LH treatment reduced TLR4 and TLR9 expression *in vivo*. **(A)** TLR4 immunohistochemical staining in colon tissue. **(B)** TLR9 immunohistochemical staining in colon tissue. **(C)** Quantitative determination of immunochemical (IHC) index of TLR4 and TLR9 positive signals. Data are shown as mean + SD (*n* = 8). **p < 0.01 vs. normal group; ^Δ^P<0.05, ^ΔΔ^P<0.01 vs. UC group; ^■^ *p* < 0.05, ^■■^ *p* < 0.01 vs. LH group.

TLR4 and TLR9 proteins were mainly expressed in the cytoplasm and membrane of the mucosal and submucosal layers of colon tissue, showing as diffuse yellow particles. Immunohistochemical staining showed that expression of TLR4 and TLR9 in both epithelial cells and inflammatory cells of colonic tissues from the UC group was remarkably upregulated compared with that of the Normal group (*p* < 0.01). After LH treatment, expression of TLR4 and TLR9 in colonic sections was notably suppressed (*p* < 0.01), and the inhibitory effect of HKL on TLR9 was enhanced after the addition of *L. acidophilus* (Figures 2A–C).

### LH Treatment Improved Gut Microbiota Diversity in UC Rats

**FIGURE 3:**
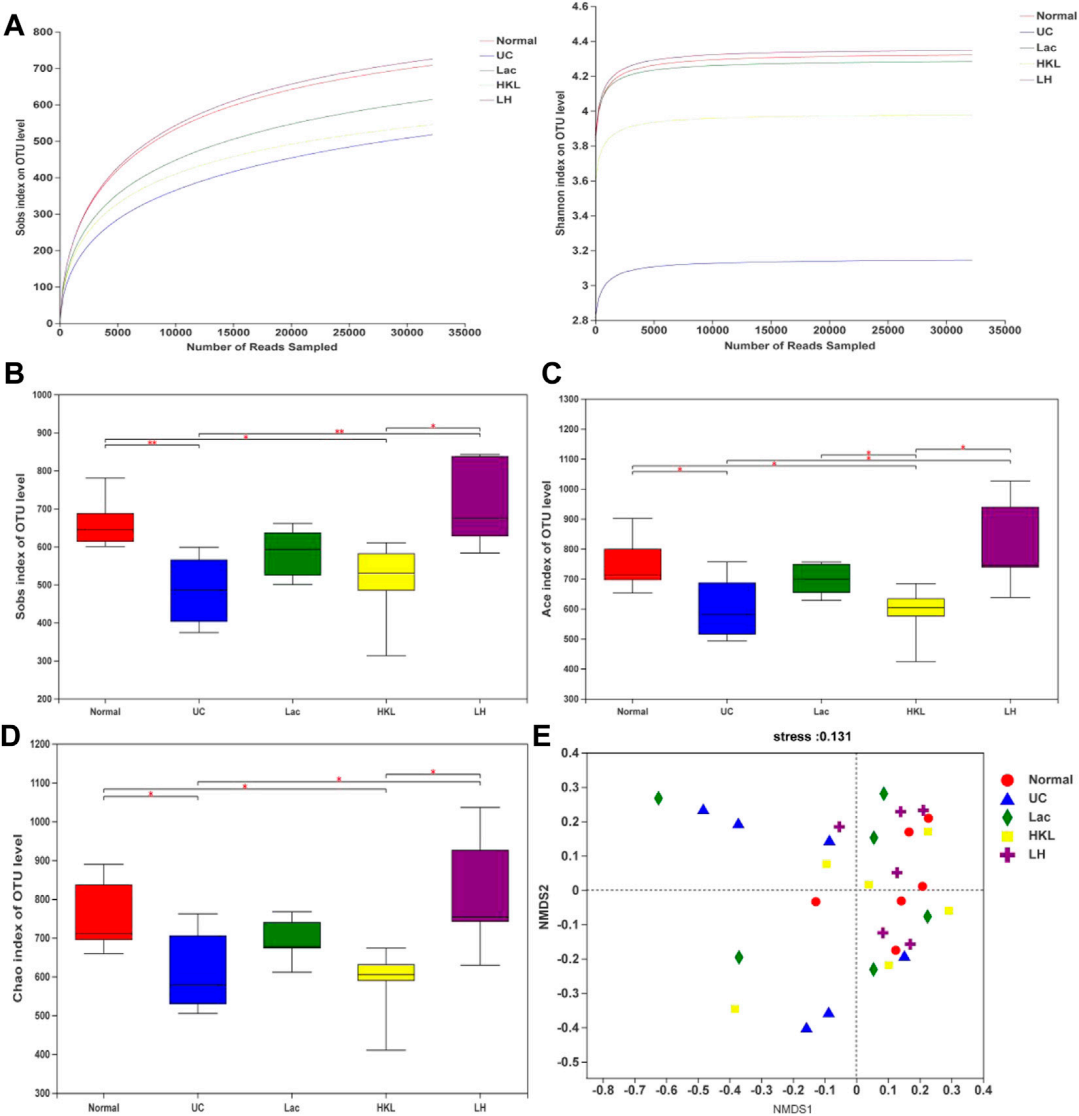
Gut microbiota diversity analysis (*n* = 5∼6). **(A)** Sobs and Shannon rarefaction curve of samples. **(B)** α-Diversity evaluated by Sobs index. **(C)** α-Diversity evaluated by Ace index. **(D)** α-Diversity evaluated by Chao index. *p < 0.05, **p < 0.01. **(E)** NMDS analysis.

We obtained 1,416,832 usable optimized raw sequences, with an average optimized sequence length of 418.61 bp. A total of 1,843 OTUs were clustered from 30 samples by bioinformatics statistical analysis. Rarefaction curves tested the data rationality of each group of the samples. A plateaued rarefaction curve of OTUs indicated that these sequencing depths covered all the species in the samples (Figure 3A). Changes in the richness and diversity of the gut microbiota were analyzed using Sobs, ACE, and Chao indices of α-diversity analysis. The α-diversity analysis (Figure 3B–D) showed a significant decrease in bacterial diversity in the UC group and an apparent increase in diversity in response to LH therapy. The results of NMDS analysis (Figure 3E) based on Bray-Cruits distance showed that the composition of intestinal flora differed among individuals, and differences were most pronounced in the UC group. Specimens from the normal and LH groups were the least discrete and the closest in distance.

### LH Treatment Modulates Gut Microbiome Composition

**FIGURE 4:**
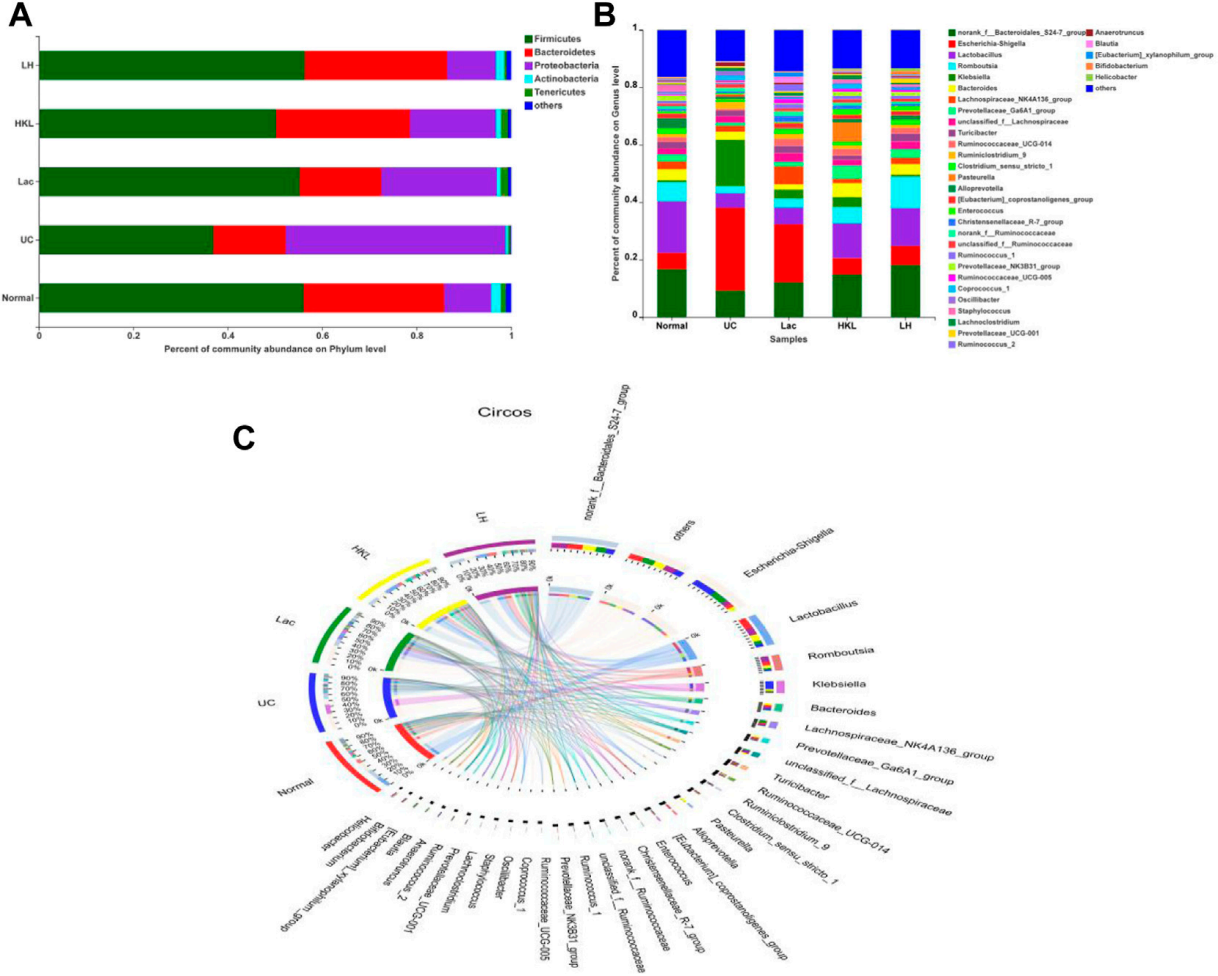
Gut microbiota composition analysis (*n* = 5∼6). **(A)** Bar plot of bacterial richness distribution at the phylum level; **(B)** bar plot of bacterial richness distribution at the genus level; **(C)** diagram of Circos analysis. Indicated are the species composition (small left semicircle), the group (color of the outer ribbon), the species (color of the inner ribbon), and the relative abundance of the species in the corresponding samples (length). The large right semicircle represents the distribution of species in samples at the taxonomic level. The outer layer ribbon represents the species, the inner ribbon color represents different groups, and the length represents the distribution proportion of the sample in a specific species.

A total of 27 phyla and 434 genera were identified. Community bar plots (Figure 4A and B) at phylum and genus levels were performed to examine whether the relative abundance of gut microbiota was associated with diversity differences. The dominant bacterial phyla in all groups were *Firmicutes*, *Bacteroidetes, Proteobacteria, Tenericutes, and Actinobacteria*. The relative abundance of Firmicutes in the normal, UC, Lac, HKL, and LH groups was 56.03, 36.94, 55.22, 50.27, and 56.29%, respectively. *Bacteroidetes* accounted for 29.73, 15.29, 17.26, 28.28, and 30.10%, respectively*. Proteobacteria* accounted for 9.99, 46.56, 24.44, 18.19, and 10.36%, respectively.

In the UC group, the abundance of *Bacteroidetes*, often associated with a healthy gut, decreased 1.94-fold, whereas *Proteobacteria*, associated with a wide variety of pathogens, increased 4.66-fold. After LH treatment, the composition of the intestinal flora was similar to that of the normal group. The distribution of dominant species in the different groups was analyzed by the Circos diagram (Figure 4C). The normal and LH groups showed similar species and abundance of flora, whereas the UC group was the most different, with *Klebsiella* and *Escherichia Shigella* being more abundant and *Lactobacillus* being less abundant.

### LH Treatment is Beneficial to the Growth of Probiotics in the Intestinal Tract and the Inhibition of Opportunistic Pathogens

**FIGURE 5:**
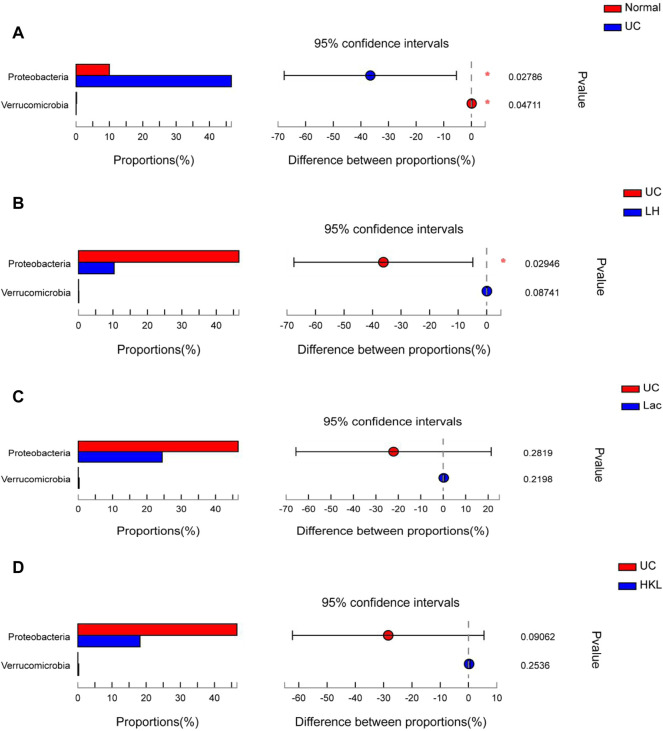
Bar plot of gut microbiota comparison at phylum level of five groups (*n* = 5∼6). **(A)** Differential phyla between the UC group and normal group. **(B)** Differential phyla between the UC group and LH group. **(C)** Proteobacteria and Verrucomicrobia comparison in the UC and Lac groups. **(D)** Proteobacteria and Verrucomicrobia comparison in the UC and HKL groups; *0.01<*p* ≤ 0.05.

A differential microbiota analysis at phylum and genus level was performed to test whether there was a significant difference between the normal and UC groups or between the LH and UC groups. In the UC group, *Proteobacteria* was more abundant than in the normal group (Figure 5A) and the LH group (Figure 5B), but the content was similar to that of the Lac and HKL groups (Figure 5C and D).

**FIGURE 6:**
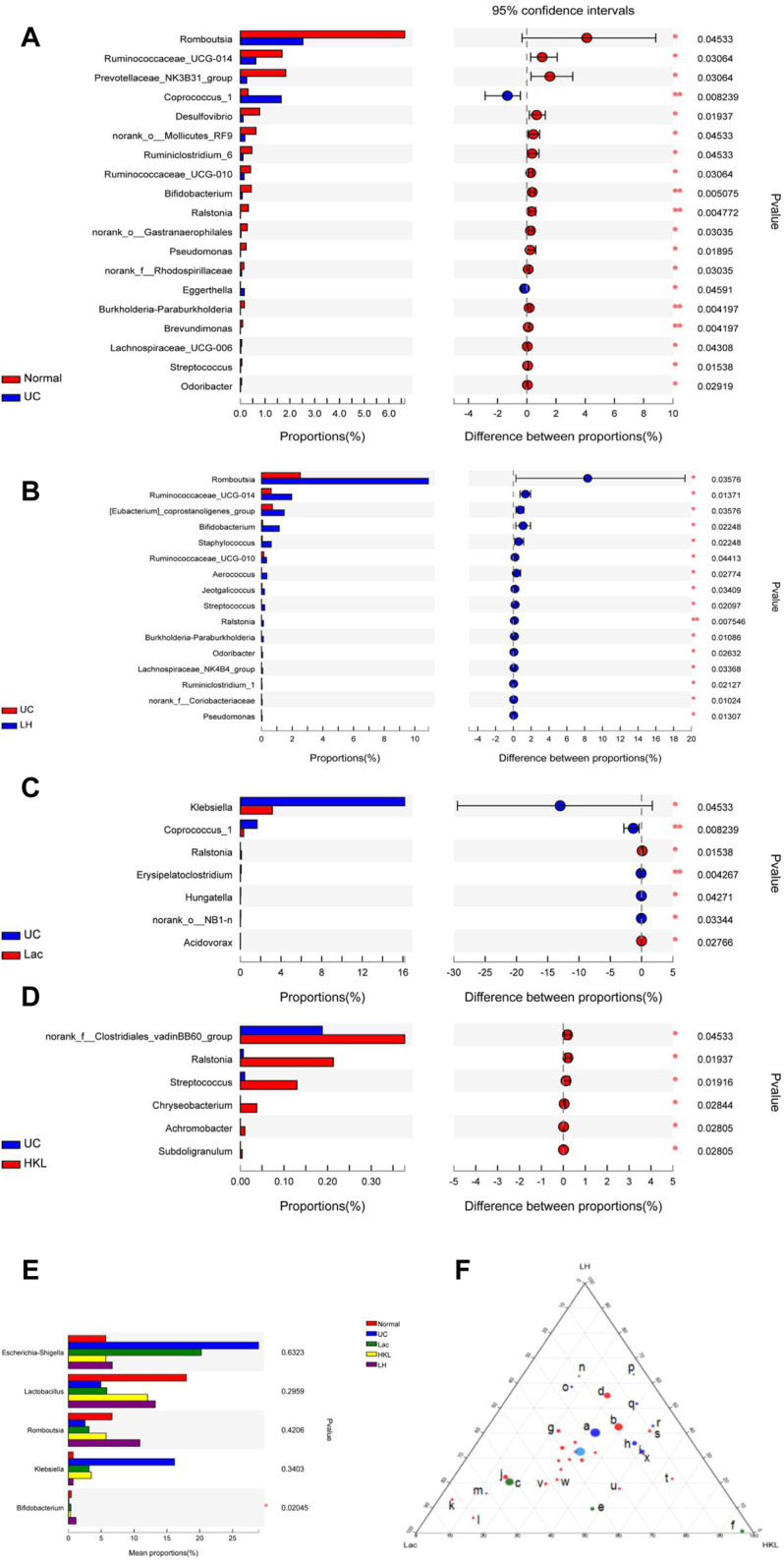
Bacterial abundance at the genus level (*n* = 5∼6). Differences between normal and UC **(A)**; UC and LH **(B)**; UC and Lac **(C)**; and UC and HKL **(D)**. **(E)** Comparison between groups of several bacteria with large change in abundance; **(F)** Ternary chart in Lac, HKL, and LH groups. (a.*bacteroidales_s24-7_grou*p; b.*lactobacillus*; c.Escherichia-Shigella; d.romboutsia; e.*klebsiella*; f.*pasteurella*; g.Turicibacter h.*bacteroides*; i.Prevotellaceae_ Ga6A1_group; j.Lachnospiraceae_NK4A136_group; k.Ruminococcus_2; l.Blautia; m.(Eubacterium)_xylanophilum; n.Bifidobacterium; o.Prevotellaceae; p.*Helicobacter*; q.Alloprevotella; r.Prevotellaceae_NK3B31_group; s.Entero coccus; t.Coprococcus_1; u.Lachnoclostridium; v.Christensenellaceae_R-7_group; w.norank_f_Ruminococcaceae; x.Prevotellaceae_Ga6A1_group).

In the UC group, the relative abundance of 17 genera such as *Romboutsia* and *Bifidobacterium* was significantly decreased relative to the normal group, whereas the abundance of *Coprococcus_1* and *Eggerthella* increased (Figure 6A). The relative abundance of several genera was higher than in the LH group: *Romboutsia, Ruminococcaceae_UCG-014, Ralstonia, Bifidobacterium, (Eubacterium]_coprostanoligenes_group, Staphylococcus, Ruminococcaceae_UCG -010, Aerococcus, Jeotgalicoccus, Streptococ*cus, Burkholderia-Paraburkholderia, Odoribacter, Lachnospiraceae _NK4B4_ group, Ruminiclostridium_1, norank_f__Coriobacteriaceae and *Pseudomonas* (Figure 6B). The relative abundance of six genera increased and Ralstonia decreased compared to the Lac group (Figure 6C), and six genera decreased compared to the HKL group (Figure 6D). At the genus level of taxonomic criteria, the UC group exhibited a high abundance of Escherichia-Shigella and *Klebsiella*, which decreased after LH treatment. The latter also resulted in an increase in Romboutsia, Bifidobacterium, and *Lactobacillus* compared to UC, HKL, or Lac groups (Figure 6E). Moreover, in the LH group, Romboutsia, Bifidobacterium, and *Lactobacillus* spp. Were abundantly clustered in the ternary diagram in the healthy intestinal mucosa (Figure 6F), indicating that LH treatment promotes the growth of probiotics in the intestine and modulates the intestinal flora in UC rats.

**FIGURE 7:**
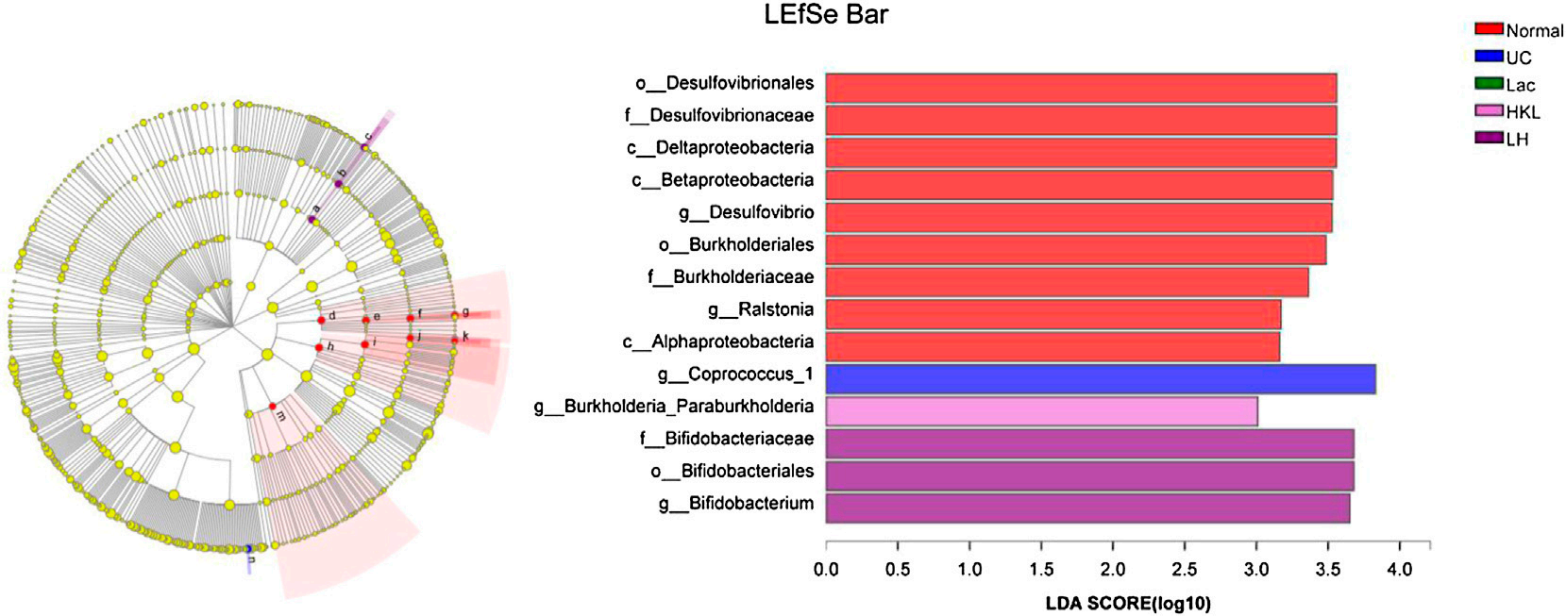
LEfSe cladogram (LDA = 3) and LDA bar chart (*n* = 5∼6). Circular cladogram for niche specialization of microbial compositions in five groups using the LEfSe analysis of the abundance patterns of bacterial taxa. Circles represent taxonomic categories of organisms from the genus level (outermost circle) to the phylum level (innermost circle). Within each given taxon, each small circle represents its lower clade. Yellow nodes indicate no statistically significant differences in a given taxon between the samples of the five groups. The size of the node is proportional to the LDA score. The links (lines) between the nodes mean hypothetical phylogenetic relationships among organisms, which can be traced back to where the lines branch off (hypothetical ancestor).

LEfSe analysis showed that many microbial taxa significantly differed between the five groups with LDA scores >3. *Coprococcus_1* was over-represented in the UC group, whereas *Bifidobacterium, Bifidobacteriales and Bifidobacteriaceae* were more abundant in the LH group (Figure 7).

### LH Treatment Correlates with *Bifidobacterium* and *Romboutsia* and Anti-Inflammatory Factors

**FIGURE 8:**
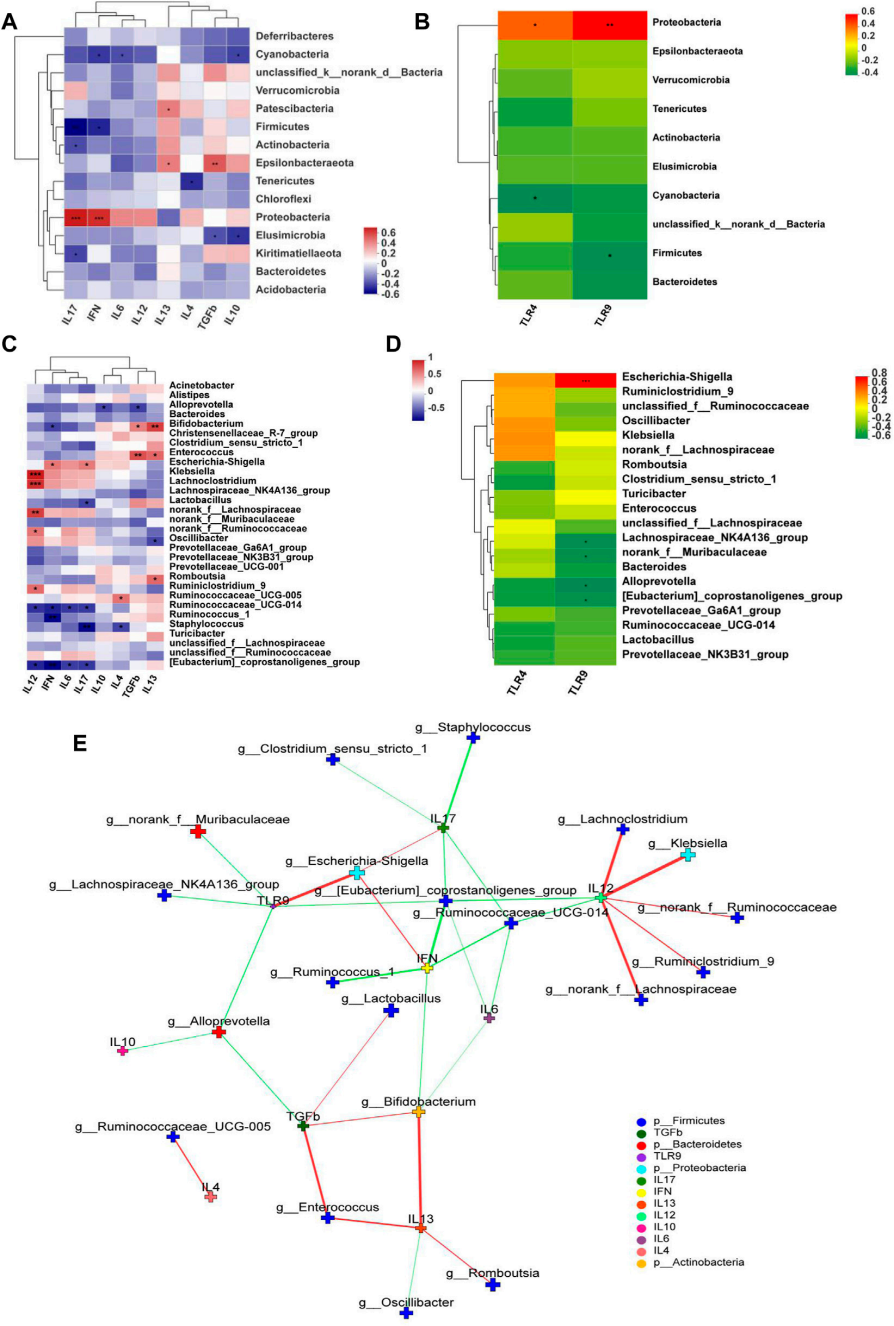
Correlation Heatmaps and network analysis (*n* = 6). Correlation heatmaps of **(A)** cytokines and gut microbiota at the phylum level; **(B)** TLRs and gut microbiota at the phylum level; **(C)** cytokines and gut microbiota at the genus level; **(D)** TLRs and gut microbiota at the genus level; **(E)** network analysis. The figure shows *p* < 0.05, correlation >0.3, the top 30 species abundance. The size of the nodes indicates the abundance of the species, and different colors indicate different species. The color of the line indicates positive (red) and negative (green) correlation.

TLR9, TLR4, IFN-γ, and IL-17 expression levels positively correlated with the abundance in *Proteobacteria*, whereas TLR9, IFN-γ, and IL-17 negatively correlated with the abundance of Firmicutes at the phylum level (Figures 8A and B). In addition, *Escherichia-Shigella* were highly correlated with TLR9, IL-17, and IFN-γ. *Bifidobacterium* was highly positively correlated with anti-inflammatory cytokines TGF-β and IL-13. *Romboutsia* was positively correlated with IL-13 (Figures 8C and D). Network analysis showed that pro-inflammatory cytokines closely correlated with *Escherichia-Shigella* and *Klebsiella* but anti-inflammatory ones correlated with *Bifidobacterium* and *Romboutsia* (Figure 8E).

### LH Treatment Restored the Balance of Serum Metabolites in UC Rats

**FIGURE 9:**
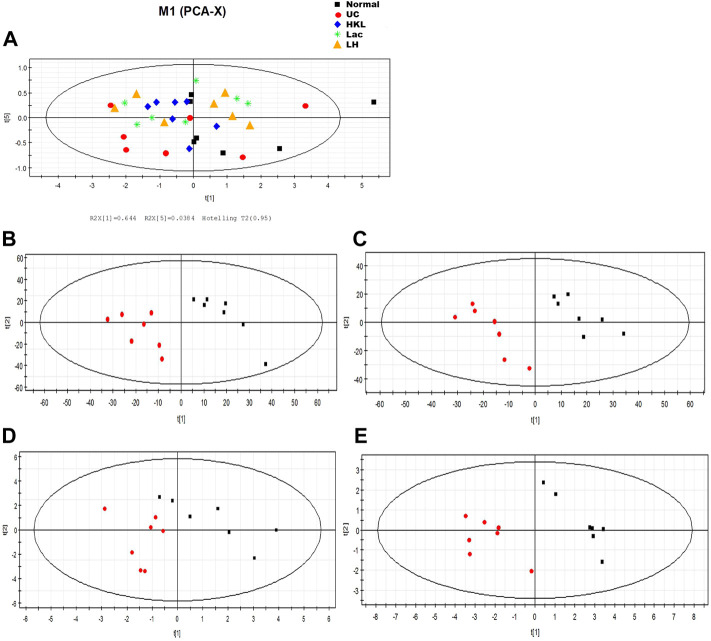
LH treatment effect on serum metabolism. **(A)** Scores plot of PCA analysis of all samples; scores plot of PLS-DA of UC vs. normal group **(B)**, HKL group **(C)**, Lac group **(D)**, and LH group **(E)**. The red marks represent UC group samples (*n* = 7).

PCA analysis showed six principal components, with model parameters R2X (cum) and R2Y (cum) of 0.958 and Q2 (cum) = 0.908. Both R2 and Q2 were relatively large, and the model fit accuracy was good (Figure 9A). To reflect the variation between group samples and the overall metabolic differences between the groups, pattern recognition analysis was performed pairwise for the different groups. The PCA score plot shows a clear separation between the UC group and the other groups, indicating a change in the serum metabolites in the UC group and an alteration of these metabolites after each intervention (Figures 9B–E).

**FIGURE 10:**
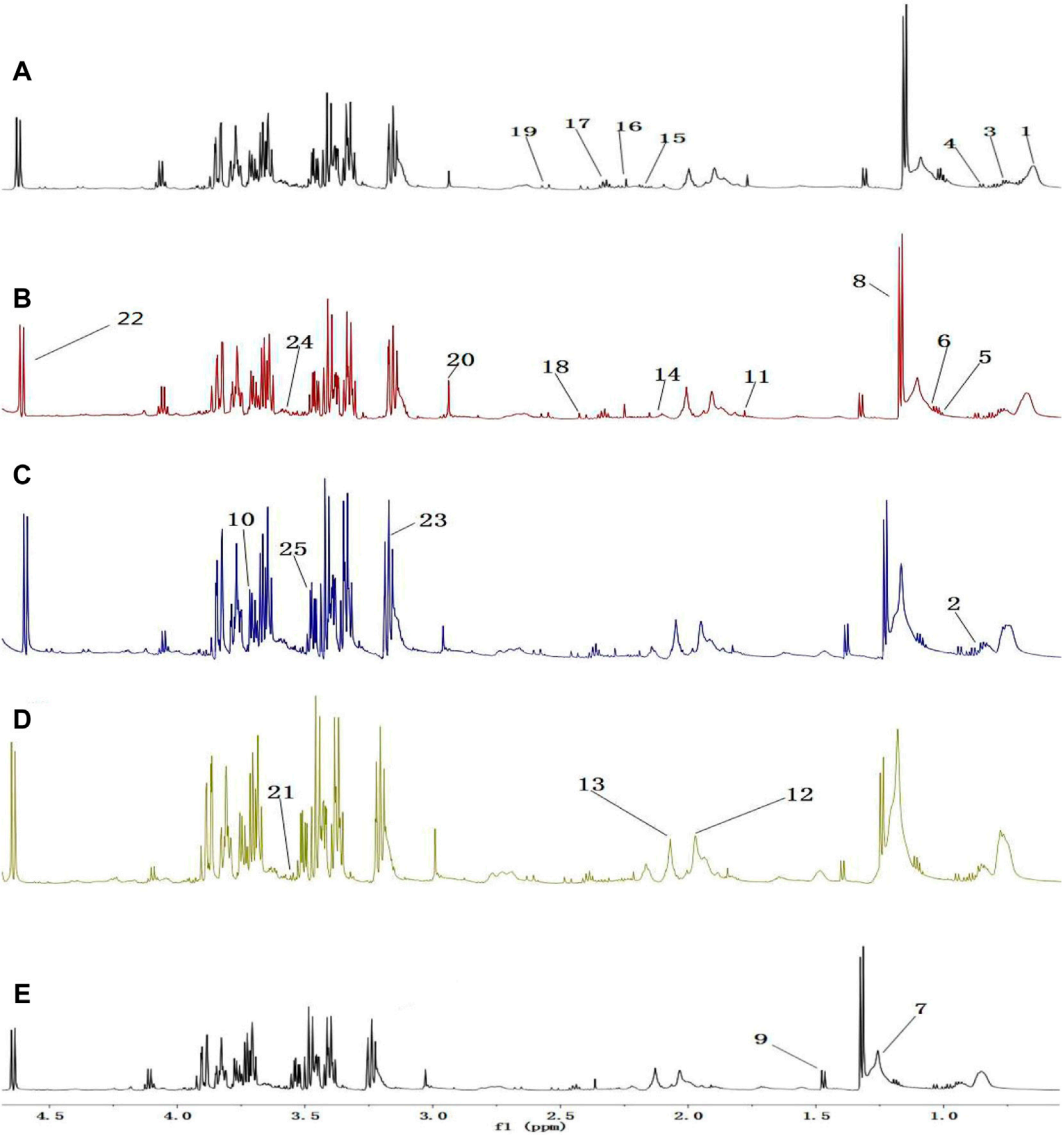
^1^H-NMR spectrum of serum (0.5–10 ppm). **(A)** Normal; **(B)** UC; **(C)** HKL; **(D)** Lac; and **(E)** LH. Numbers represent the peaks of metabolites: 1. VLDL; 2. L-Isoleucine, 3. Leucine; 4. Valine; 5.3-hydroxybutyric acid; 6. Lipids; 7. LDL, 8. Lactic acid, 9. Alanine, 10. Lysine; 11. Acetate; 12. Glycoprotein; 13. Methionine; 14. Acetone; 15. Acetoacetic acid; 16. Pyruvic acid; 17. Carnitine; 18. Methylamine; 19. Citric acid; 20. Creatine; 21. Inositol; 22. Glucose; 23. Taurine; 24. Choline; 25. Glycine.

A comparison between representative NMR spectra of specimens from the five groups is shown in Figure 10. The serum metabolome can provide the functional readout of the gut microbiome. Thus, H-NMR metabonomic analysis was used to compare the metabolic characteristics among the five groups. The UC group showed enrichment and depletion in several metabolites when compared to the normal group (Table 1). Differences were also observed when compared to the HKL, Lac, and LH groups (Table 2). These altered metabolites were mainly involved in carbohydrate, amino acid, and lipid metabolism.

**TABLE1:** Differentially expressed metabolites in the normal and UC groups.

Metabolites	Chemical shift	VIP	Trend
3-hydroxybutyric acid	1.17(d)	1.73	↓
Lipids	1.19(**d**)	1.57	↓
LDL	1.25(m)	4.26	↓
Lactic acid	1.32(**d**)**4.12**(**q**)	2.74	↑
Alanine	1.47(**d**)	1.40	↓
Acetate	1.91(**s**)	1.94	↓
Acetone	2.22(m)	1.51	↓
Methyl amine	2.53(s)	1.12	↑
Citric acid	2.65(d)2.76(d)	1.46	↓
Creatine	3.03(**s**)**3.93**(**s**)	2.49	↑
Choline	3.21(s), 3.66(m)	1.17	↑
Inositol	3.54(**dd**)3.64(dd)4.05(t)	1.80	↑
Glycine	3.55(**s**)	2.53	↑
Glucose	4.64(**d**)**5.22**(**d**)	4.05	↓

**TABLE 2:** Differentially expressed metabolites in the UC and drug intervention groups.

Metabolites	Chemical shift	VI*P score*/Trend		
		UC/HKL	UC/Lac	UC/LH
Isoleucine	0.92(t)	-	2.00↑	-
LDL	1.25	-	-	2.11↑
Lactic acid	1.32(**d**)**4.12**(**q**)	2.70↓	1.64↓	2.79↓
Alanine	1.47(**d**)	1.21↑	-	1.30↑
Acetate	1.91(**s**)	1.42↑	-	1.42↑
Creatine	3.03(**s**)**3.93**(**s**)	1.23↓	1.34↓	1.92↓
Choline	3.21(s), 3.66(m)	1.07↑	-	-
Taurine	3.25(**t**)**3.41**(**t**)	5.43↑	-	4.92↑
Inositol	3.54(**dd**)3.64(dd)4.05(t)	1.57↓	2.08↓	2.02↓
Glycine	3.55(**s**)	2.53↓	1.26↓	1.91↓
Glucose	4.64(**d**)**5.22**(**d**)	2.87↑	2.89↓	1.84↑

### LH Treatment can Restore the Metabolic Disorder in UC Rats by Regulating Intestinal Microorganisms

**FIGURE 11:**
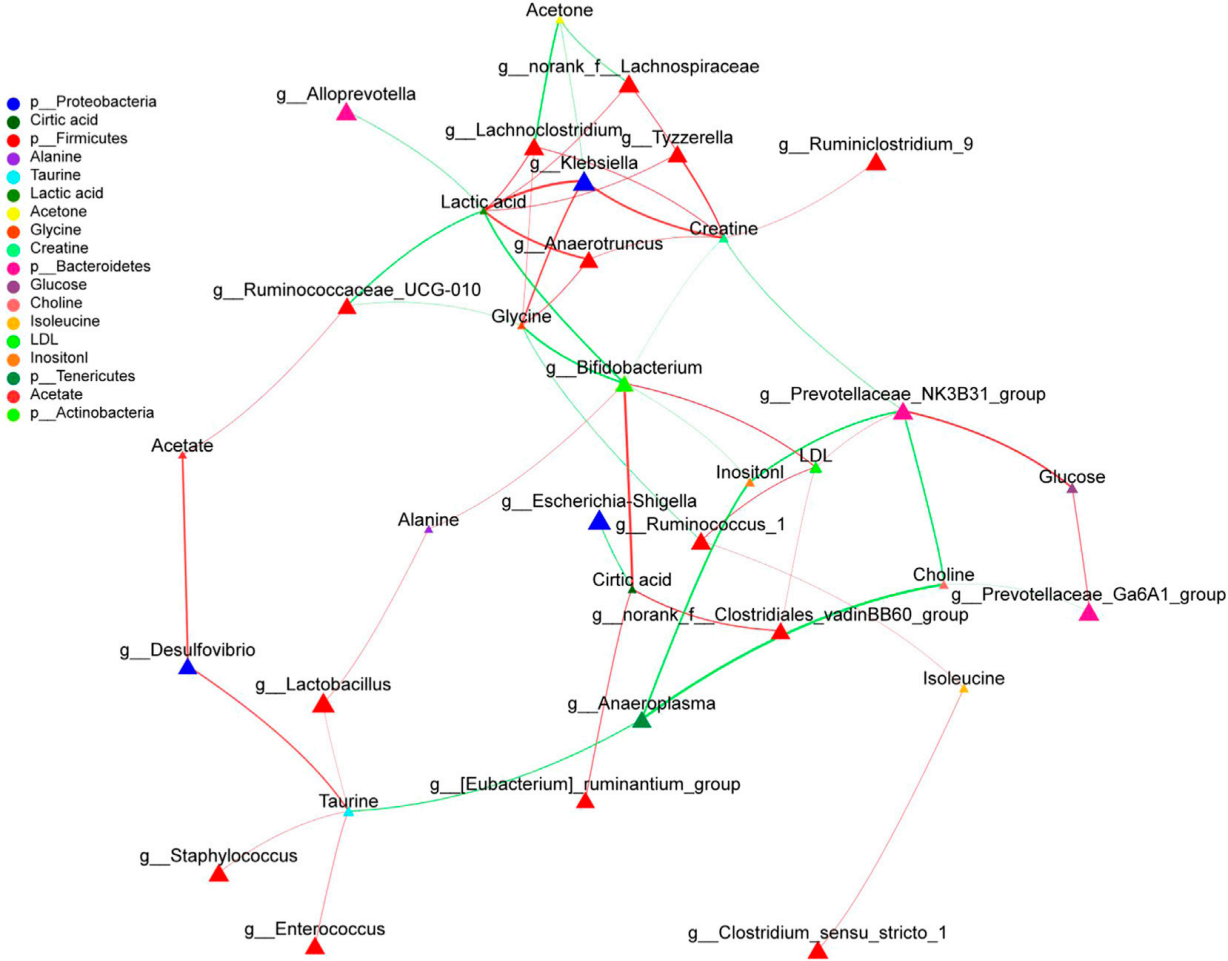
Network analysis of the abundances of gut microbiota and serum metabolites.network analysis. The figure shows *p* < 0.05, correlation >0.5, the top 50 species abundance. The size of the nodes indicates the abundance of the species, and different colors indicate different species. The color of the line indicates positive (red) and negative (green) correlation.

We analyzed the potential correlations between the abundance of gut microbiota (at the genus level) and serum metabolites (Figure 11; Table 3). Bacterial species formed strong and broad co-occurring relationships with serum metabolites. *Bifidobacterium, g_prevotellaceae_NK3B31_group, g_klebsiella, g_lachnoclostridium, g_Ruminococcus-1, g_Anaerotruncus, g_Ruminococcaceae_ UCG_010, and g_Anaeroplasma* were related to the changes of multiple metabolites in the serum.

**TABLE 3:** Correlations between gut microbiota and serum metabolites.

Gut Microbiota		Metabolites	R	P
Phylum	Genus			
*Proteobacteria*	*Klebsiella*	Glycine	0.703	0.0108
		Lactic acid	0.789	0.0023
		Creatine	0.747	0.0053
		Acetone	−0.596	0.0407
*Actinobacteria*	*Bifidobacteium*	Creatine	−0.668	0.0176
		Lactic acid	−0.600	0.0393
*Firmicutes*	*Lachnoclostridium*	Lactic acid	0.650	0.0220
		Glycine	0.600	0.0393
		Creatine	0.635	0.0267
		Acetone	−0.721	0.0081
	*Ruminococcaceae-UCG-014*	Creatine	−0.759	0.0042
		Lactic acid	−0.809	0.0014
	*Lactobacillus*	Alanine	0.592	0.0426
	*Ruminococcus*	LDL	0.642	
		Glycine	−0.624	0.0302
	*Ruminococcus*	LDL	0.642	0.0244
	*Tyzzerella*	Creatine	0.659	0.0197
		Lactic acid	0.623	0.0306
	*Ruminiclostridium_9*	Creatine	0.596	0.0409
*Bacteroidetes*	*prevotellaceaeNK3B31*	Creatine	−0.606	0.0367
		Glucose	0.739	0.0061
		Choline	−0.695	0.0122
		Inositol	−0.713	0.0093
	*prevotellaceaeGa6A1*	Creatine	−0.577	0.0496
		Glucose	0.643	0.0240
		Choline	−0.582	0.0469
	*Alloprevotella*	Lactic acid	−0.618	0.0321
		Creatine	−0.579	0.0486

Note: R represents the correlation coefficient value.

## Discussion

The pathogenesis of UC involves intestinal flora disorder and intestinal immune abnormalities (Frank et al., 2011; Nishida et al., 2018). Therefore, intestinal microbiota regulation and anti-inflammatory therapy may play an essential role in UC treatment. TCM has been broadly applied for treating UC in China for a long time due to its safety and efficacy in UC. In this study, *L.acidophilus* and HKL were used to treat UC in rats, and the mechanism of the LH treatment was explored from the perspectives of intestinal flora, immunity, and metabolism.

The innate immune system in the gut is the first line of defense against various bacterial pathogens. In UC patients, the weak innate immune system leads to the accumulation of bacterial antigens that stimulate the acquired immune system to produce a cascade of inflammation (Wu and Zhou, 2021). This causes an imbalance between pro- and anti-inflammatory factors. In particular, various pro-inflammatory mediators increase, causing local and systemic inflammation (Head and Jurenka., 2003; Zhang et al., 2006). Indeed, we have shown that in the UC group, expression levels of IL-12, IFN-γ, IL-6, IL-17, TLR4, and TLR9 were upregulated, whereas the protein expression level of anti-inflammatory factor TGF-β was downregulated. IL-12 and IFN-γ are important UC-related inflammatory cytokines that can induce the differentiation of CD4+ T cells into Th1 cells (Rowan et al., 2010; Andoh et al., 2011; Workman et al., 2014). The interaction between Th1 cells, IFN-γ, and IL-12 enhances the differentiation ability and function of Th1 cells. This leads to the imbalance in Th1/Th2 cells, resulting in excessive expression of pro-inflammatory factors that induce inflammation. TGF-β is an inflammatory cytokine, co-required for the differentiation of Th17 and Treg cell subsets, which has immunosuppressive and anti-inflammatory effects (Sara and Pizarro, 2015). The observed low concentration of TGF-β in UC colon tissue leads to the secretion of IL-17, IL-6, and other pro-inflammatory factors. This reduced TGF-β expression may be beneficial to the differentiation of naive T cells (Th0 cells) into Th17 cells. In the LH group, expression levels of IL-4, IL-13, and TGF-β were significantly upregulated, indicating an anti-inflammatory effect. TLR9 protein is rarely expressed in colonic epithelial cells, whereas upregulation of TLR9 is observed during inflammation in mucosal infiltrating cells (Sánchez-Muñoz et al., 2011; Pedersen et al., 2005), consistent with our results. In the LH group, the expression levels of TLR4 and TLR9 were significantly downregulated. Pathogenic bacterial DNA induces TLR9 expression *in vitro*, while dendritic cells respond to symbiotic bacterial DNA by TLR9 signaling to inhibit Treg differentiation in the intestinal tract (Hall et al., 2008). These results indicate that LH treatment reduced TLR9 protein expression and Treg cell differentiation, increased TGF-β and IL-10 protein expression, and repaired colon mucosal injury, restoring the intestinal microflora balance. Our previous study showed that Treg cell count increased, and Th17 cell count decreased in the blood of UC rats after LH intervention (Kasimujiang et al., 2021).

The intestinal flora interacts with the host immune system, which determines the structure and function of intestinal flora (Hans et al., 2000; Tlaskalova-Hogenova et al., 2004). Imbalance of the intestinal flora causes proliferation of opportunistic pathogens, induces the expression of related genes through TLRs-mediated signal transduction pathway, triggers and regulates innate immunity, induces acquired immunity, and activates the inflammatory response of the intestinal mucosa (Autenrieth, 2017; Sartor and Wu., 2017).

Species diversity in the gut is an important indicator of intestinal health, and the intestinal flora of a healthy gut is always in balance. It has been proposed that reduced bacterial diversity in rectal mucosa samples from UC patients is associated with intestinal inflammation (Niskawa et al., 2009; Zuo et al., 2019). If the diversity of intestinal flora decreases, its ability to resist environmental change is weakened (Sheehan et al., 2015; Sommer et al., 2017; Franzosa et al., 2019; Nishida and Ochman., 2019). We have shown that in the UC group the community richness of bacteria and Sobs, ACE and Chao indices were markedly decreased. In contrast, in the LH group, these indices increased, indicating a richer gut microbiota that allowed compensation of functionally deficient species with other similar species. Herein we found that the common dominant bacteria in the five groups of rats were *Firmicutes*, *Proteobacteria, Bacteroidetes, Actinomycetes, and Tenericutes*. *Firmicutes* and *Bacteroidetes* were more abundant (85.82%) in the normal group, with *Firmicutes* being more abundant than *Bacteroidetes,* as consistent with previous reports (Lee et al., 2018).

*Proteobacteria* are currently a dominant group that increases under conditions of endotoxemia, metabolic disturbance, and persistent inflammation, which may be a marker of microbial instability (Qin et al., 2012; Slingerland et al., 2017). *Proteobacteria* are the dominant bacteria in the intestinal tract of UC rats (McLaughlin et al., 2010; Shah et al., 2016). In the present study, they were 4.68 times more abundant than in the normal group and were positively correlated with the protein expression levels of IL-17, IFN-γ, TLR4, and TLR9. Microbial adhesion to intestinal epithelial cells (ECs) is the key to induce Th17 cells, and p*roteobacteria* adhere to intestinal epithelium (Atarashi et al., 2015). Thus, the increased abundance of *proteobacteria* in UC rats may induce the adhesion of enteric adhesive *EScoli* (EAEC) to EC, promoting the differentiation of Th17 cells. The latter can upregulate T-bet and produce IFN-γ, resulting in the production of IL17/IFNγ double-positive cells Th1/Th17 with increased secretion of IL-17 and IFN-γ at the intestinal mucosa (Lee Y. K. et al., 2009). TLRs activate and regulate the maturation of dendritic cells (DCS) and induce the proliferation and differentiation of Th1 and Th2 cells (Lu et al., 2018). Therefore, *proteobacteria* induction of Th1 cells through TLR9 recognition to promote IFN-γ release may be one of the reasons for the imbalance of cytokines in UC. TLR9 also has a role in innate host resistance to Gram-negative bacteria such as *Klebsiella* (Bhan et al., 2007; Sjolinder et al., 2008). *Proteobacteria* abundance was similar in UC, Lac, and HKL groups but lower in the LH group. This suggests that LH treatment was effective in inhibiting *Proteobacteria* and may be linked to a therapeutic effect. In our study, *Escherichia-shigella* and *Klebsiella* in *proteobacteria* were more abundant in UC rats. *Klebsiella* is usually associated with the development of cancer (Takamura et al., 2011; Stecher et al., 2013), whereas *Escherichia-shigella* is the most typical pathogen. Although the abundance of these two bacteria in the intestinal tract of UC rats was significantly higher than in the normal group, no differences were observed between UC and LH groups due to the large intra-group differences. These results indicate that individual differences or the degree of the ulcer may affect the intestinal abundance of these two bacteria in UC patients. Our study found that *Escherichia-Shigella* was positively correlated with IL-17 and IFN-γ, and *Klebsiella* was positively correlated with IL-12, suggesting that they promote UC by affecting pro-inflammatory cytokines and leading to the disorder of cytokine balance. After LH treatment, the abundance of *Klebsiella* decreased, and pro-inflammatory cytokines were downregulated.

Under *Actinobacteria*, *Bifidobacterium* helps prevent colitis and maintain intestinal homeostasis (O’Mahony et al., 2001)and produces a broad spectrum of nutritional and antibacterial substances and acidic metabolites that reduce the pH level of the intestinal tract, thereby inhibiting or destroying pathogens. In addition, it can bind to the surface of intestinal epithelial cells through teichoic acid adhesion, forming a membrane barrier and inhibiting the migration, invasion, and colonization by endogenous and exogenous intestinal pathogens and triggering immune responses *in vivo* (Nielsen and Rask-Madsen, 1996; Zhiwei et al., 2016). We have shown that *Bifidobacterium* decreased in the UC group, suggesting that TNBS reduced the content and colonization ability of *Bifidobacterium.* The imbalance of the bacterial community in TNBS-induced UC is consistent with previous reports (Becker et al., 2015; Yun et al., 2020). *Bifidobacterium* can repair the impaired intestinal epithelial barrier function and reduce IFN-γ secretion in the intestinal mucosa (Pozo-Rubio et al., 2011). In our study, *Bifidobacterium* content was negatively correlated with IFN-γ protein expression level and positively correlated with TGF-β and IL-13. After LH treatment, *Bifidobacterium* was significantly enriched, suggesting that it may play a role in repairing intestinal mucosal injury caused by UC by inhibiting the secretion of pro-inflammatory factors and promoting the secretion of anti-inflammatory factors. LH treatment also increased the abundance of *Lactobacillus. Bifidobacterium* and *Lactobacillus* are the main intestinal probiotics, promoting absorption of nutrients and promoting the balance of the intestinal normal flora and reducing the occurrence of gastrointestinal diseases, among other positive effects (Chen, 2011). *Romboutsia*, like *Lactobacillus,* belongs to *Firmicutes* (Han et al., 2011) and is part of the normal flora of the human digestive and respiratory tracts and may be lost in an undernourished environment (Caplan et al., 1999). We observed a decrease in the abundance of *Romboutsia* in the UC group and an increase in the LH group. This suggests a beneficial effect of LH treatment in the intestinal tract, which promotes a balance in the intestinal environment.

**FIGURE 12:**
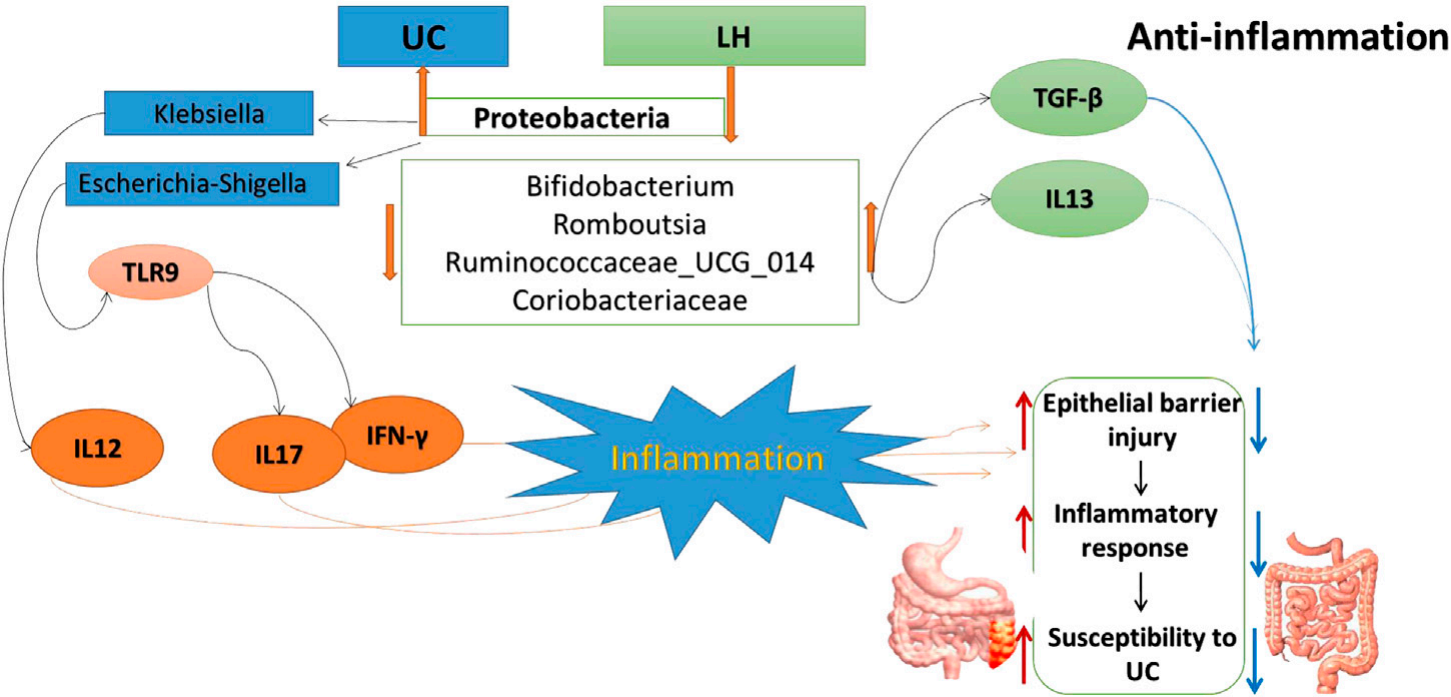
Possible partial mechanisms of UC pathogenesis and LH treatment for UC.

*Firmicutes* promote the absorption of calories and various nutrients in food. In the intestinal tract of normal children, the content of *Firmicutes* is 9.6 times higher than in malnourished children (Wu and Jiang., 2019). In addition, increased intestinal *Firmicutes* and *Actinomycetes* in breastfed infants may be associated with a lower incidence of gastrointestinal infections (Duijts et al., 2010; Azad et al., 2013). In our study, *Firmicutes* in the UC group were significantly reduced and correlated with IL-17, IFN-γ, and TLR9. Thus, in UC, the increase of pro-inflammatory cytokines expression appears to be linked to the decrease of *Firmicutes*. After LH treatment, pro-inflammatory cytokines were reduced, and the number of *Firmicutes* increased, further supporting a link between *Firmicutes* and intestinal inflammation. In all, the occurrence of UC may be related to the excessive growth of opportunistic pathogens that activate TLR9 and increase the expression of pro-inflammatory factors. LH intervention is beneficial to promoting *Bifidobacterium’s* growth and other probiotics in the intestinal tract due to its microflora regulation. HKL has a strong anti-inflammatory effect in LH treatment, so LH treatment plays a role in treating UC through anti-inflammatory and regulation of intestinal flora (Figure 12).

The resident bacteria *Bacteroidetes* are responsible for maintaining mucosal homeostasis and providing protection for the mucosal barrier. Their loss is associated with dysregulation of the intestinal flora. In the colon tissue of UC rats, we observed a decrease in the abundance of *Bacteroidetes*. This may cause weakened immune regulation and an unbalanced intestinal flora. After LH treatment, *Bacteroidetes* content was close to that of the normal group, suggesting that LH treatment promotes strengthening of the immune regulation of intestinal mucosa. Intestinal butyrate and polyunsaturated fatty acids can protect the intestinal mucosa and provide energy for the colon mucosa, inhibit the signaling pathway of pro-inflammatory cytokines, and reduce the secretion of pro-inflammatory cytokines (Lu SHEN., 2011; Morgan et al., 2012). SCFA promotes the expansion of regulatory T cells in the colon, thus promoting the self-healing of the intestinal tract to relieve UC symptoms and prevent its recurrence (Kostic et al., 2014; Goverse et al., 2017). Indeed, SCFAs such as acetate, propionate, and butyrate are consumed as anti-inflammatories in UC patients (Sartor and Wu., 2017; Schirmer et al., 2018). Certain *Ruminococcaceae* genera can consume hydrogen to produce acetate, which is subsequently used by *Roseburia* to produce butyrate (Paradaenegas et al., 2019). In the UC group, we found a reduction of butyrate-producing bacteria such as *Bacteroidales S24-7, Romboutsia, Ruminococcaceae_UCG_014, and Ruminococcaceae_UCG_010*, suggesting that with the decrease of SCFA producing bacteria in UC rats, colonic epithelial cells have insufficient energy, resulting in decreased resistance of colonic mucosa to external stimulation and increased UC. As energy decreases, *bacteroides* protecting the intestinal mucosal barrier also decrease (Mera 2017). After LH treatment, SCFA producing bacteria, e.g., *Ruminococcaceae_UCG_014, Ruminococcaceae_UCG_010, Romboutsia,* and *Norank-F- coriobacteriaceae* increased significantly. SCFA can prevent colitis by regulating Treg cell production and enhancing the antibacterial activity of macrophages (Smith et al., 2013; Fernando et al., 2016). Thus, LH treatment may address the energy deficiency in colonic epithelial cells of UC rats by increasing SCFA producing bacteria. In the UC group, *Ruminococcaceae_UCG_014* also decreased, consistent with the literature (Wang et al., 2018). *Ruminococcaceae_UCG_014* negatively correlated with IFN-γ, IL-6, IL-12, and IL17. In the LH group, *Ruminococcaceae_UCG_014* increased, and this can lead to energy availability for intestinal epithelial cells by increasing the production of SCFA and inhibiting pro-inflammatory factors secretion.

The intestinal flora plays an important role in facilitating nutrient digestion and absorption, metabolism, and maintaining host immunity and intestinal barrier balance (Van de Guchte et al., 2018). Tissues obtain energy mainly through aerobic oxidation and pyruvate is an end product of glycolysis and in the absence of oxygen is dehydrogenated to lactic acid. In the UC group, glucose and citric acid content in serum decreased, whereas lactic acid increased, suggesting an enhancement of the anaerobic oxidation and a state of energy deficiency. This generation of lactic acid is consistent with a recent report (BjerrumJT et al., 2017). In the UC group, plasma lactic acid concentration increased, and lactic acid content was negatively correlated with *Alloprevotella*, an SCFA-producing bacteria. This indicates insufficient energy supply to the intestinal epithelial cells leading to increased lactic acid concentration. With the large amount of pyruvate entering the cytoplasm to produce lactic acid, the pyruvate entering the mitochondria to produce acetyl-coA will decrease, and the sugar aerobic oxidation pathway will be relatively inhibited, resulting in the decrease of tricarboxylic acid cycle intermediates such as citric acid. In the LH group, lactic acid content decreased, and glucose and alanine increased, which suggests that LH treatment returns energy metabolism to normal; pyruvate re-enters the aerobic oxidation pathway, and lactic acid content decreases. In the UC group, glucose concentration decreased, and inositol content increased. *Prevotellaceae_Ga6A1_group* and *Prevotellaceae_NK3B31_group* positively correlated with glucose, whereas the latter negatively correlated with inositol, suggesting a role of *Prevotellella* in energy metabolism.

Low-density lipoprotein (LDL) can be converted into bile acids in the intestine to aid lipid digestion and absorption. Some LDL is synthesized by the liver and secreted directly into the blood. Detoxification of lipoprotein can prevent inflammation caused by endotoxins (Harris et al., 2002). In the UC group, serum LDL levels decreased, which may be a manifestation of the gradual onset of systemic inflammatory response. LDL can be caused by abnormal liver metabolism and impaired LDL synthesis. Choline, as a product of glycerophospholipid metabolism, is an essential nutrient. A disrupted choline metabolism will interfere with the stability of cell membrane and signal transmission (Michel et al., 2006; Glier et al., 2014). An increase in choline metabolites was associated with drug-induced cell membrane damage (Grifin et al., 2001). In the UC group, the increased serum choline content may be related to the chemical damage of intestinal mucosa caused by TNBS, resulting in the decline of intestinal barrier function. The decrease of LDL and the increase of choline further suggested that TNBS/ethanol chemical stimulation induces lipid peroxidation and damages the integrity of the cell membrane. In the LH group, serum LDL levels increased, and glycine and choline decreased. These changes suggest a decline in lipid peroxidation and a regulatory effect on lipid metabolism. Interestingly, choline content was higher when HKL was used alone than in the UC group, with no significant difference in LDL levels. These results suggest that the LH treatment is better than a single treatment on the metabolic pathway. Ketone body is a way of the energy output of liver cells, including 3-hydroxybutyric acid, acetone, and acetoacetic acid. In this study, the contents of 3-hydroxybutyric acid and acetone in the UC group decreased, which may be caused by accelerating the decomposition of ketone bodies generated in the liver to relieve the serious energy deficiency of the body.

Amino acids and their metabolites play an important role in maintaining body homeostasis and are important regulators of cell metabolism, cell growth, and proliferation (Brand, 1985; Barbul, 1986; Garlick, 2005; Grohmann and Bronte, 2010). Dietary protein or amino acid deficiencies are thought to impair immune function and increase human susceptibility to infection (Li et al., 2007). UC patients have abnormal amino acid metabolism in plasma, serum, and colon tissues (Bjerrum et al., 2015). In the UC group, serum alanine content decreased while glycine content increased. Alanine may be metabolized to pyruvate by alanine aminotransferase (ALT), entering the catabolic pathway to assist energy production. Glycine is a glycogenic amino acid that reduces the production of free radicals, regulates the production of inflammatory mediators, and protects tissue cells. Thus, the increase in glycine in the UC group may be used to compensate for the damage to tissue cells caused by oxygen free radicals generated by lipid peroxidation. Creatine is also synthesized from glycine. Glucogenic amino acids can be converted into glucose. The latter produces energy through oxidative decomposition and participates in tissue repair and blood sugar regulation. Plasma creatine levels increase in infectious diseases resulting in lower ATP (Jabs et al., 1998), consistent with our results. In the UC group, creatine content negatively correlated with *Alloprevotella, prevotellaceae_Ga6A1_group, prevotellaceae_NK3B31_group* and the abundance of *Prevotellaceae _NK3B31_ group* was lower. *Prevotella* plays a role in carbohydrate fermentation, producing exogenous SCFA (Salminen et al., 1998; Pouteau et al., 2003; Zhang et al., 2009). In the UC group, *Prevotella* decreased in the colon, whereas creatine content increased in plasma. In this group, the intestinal epithelial cells were deficient in energy sources. This insufficient energy supply may have led to an increase in creatine content, compensating for the decrease in ATP.

Organs that consume more energy use anaerobic oxidation of sugar, which leads to the increase of lactic acid. Glycine is the raw material for creatine synthesis. In the LH group, glycine and creatine decreased, whereas no differences were observed in β -hydroxybutyric acid and acetone between UC and LH groups, indicating that energy supply recovery was not complete after LH treatment. This suggests that the regulation of metabolism in UC rats is time-dependent.

## Conclusion

Our study highlighted the anti-inflammatory effects of LH treatment in TNBS-induced colitis. Treatment with *L. acidophilus* and HKL suspension improved the gut microenvironment and exerted anti-inflammatory effects in rats with TNBS-induced colitis. LH treatment suppressed the TLR9 expression, resulting in the downregulation of inflammatory cytokines and upregulation of anti-inflammatory cytokines. The increase of *Klebsiella* may be an essential signal of metabolic disorder in UC rats and is positively correlated with lactic acid, creatine, and glycine levels. The combined intervention inhibited the growth of *Klebsiella* and affected the metabolism of lactic acid and creatine. Our study provides experimental evidence that a combination of TCM and probiotics may be a potential candidate for UC treatment.

The molecular mechanism of LH treatment for UC was explored in this study, but this work still needs further research on the cellular level, signaling pathways, and side effects on the body. This work may provide a clear basis for the pathogenesis of UC and LH treatment.

## Data Availability

The datasets presented in this study can be found in online repositories. The names of the repository/repositories and accession number(s) can be found below: https://www.ncbi.nlm.nih.gov; PRJNA819066.
